# Do Male Desert Gobies Compromise Offspring Care to Attract Additional Mating Opportunities?

**DOI:** 10.1371/journal.pone.0020576

**Published:** 2011-06-08

**Authors:** Nicholas Symons, P. Andreas Svensson, Bob B. M. Wong

**Affiliations:** School of Biological Sciences, Monash University, Melbourne, Victoria, Australia; University of Western Ontario, Canada

## Abstract

Males often play a critical role in offspring care but the time and energy invested in looking after young can potentially limit their ability to seek out additional mating opportunities. Recent studies, however, suggest that a conflict between male parental effort and mating effort may not always be inevitable, especially if breeding occurs near the nest, or if parental behaviours are under sexual selection. Accordingly, we set out to experimentally investigate male care and courtship in the desert goby *Chlamydogobius eremius*, a nest-guarding fish with exclusive paternal care. Despite courtship occurring near the nest, we found that when egg-tending males were given the opportunity to attract additional females, they fanned their eggs less often, engaged in shorter fanning bouts, and spent more of their time outside their nests courting. Our findings highlight the importance of understanding the circumstances under which reproductive tradeoffs are expected to occur and how these, in turn, operate to influence male reproductive decisions.

## Introduction

Looking after young can be a costly endeavour [1], [2], [3]. In many species, males often contribute substantially to offspring care, but both the time and energy invested by males can potentially limit their ability to seek out additional mating opportunities (reviewed in [4]). Since males are expected to enhance their reproductive success mainly by maximising their number of mating partners [5], such limitations can have important consequences for male fitness, as shown, for example, in fairy martins (*Hirundo ariel*) [6] and collared flycatchers (*Ficedula albicollis*) [7].

Whilst it is true that temporal and energetic limitations can often result in conflict between mating and parental effort, it is important to realize that such tradeoffs are not always inevitable (reviewed in [8]). For example, in many polygynous species with male-only care, continued breeding often takes place at the same location where males are looking after their young (e.g. nesting sites). Here, it is predicted that males who are tending to the needs of existing offspring can also attract additional mating opportunities without having to compromise their parental responsibilities [9]. Further, females are sometimes able to select mates by directly observing the quality of care provided [10], or by using cues that reliably reflect a male's parental abilities (e.g. the presence of eggs already in the nest; [11]). In this regard, males might even increase their parental effort in the presence of choosy females [10], [12].

Here, we set out to experimentally examine the relationship between male parental and mating effort in the desert goby (*Chlamydogobius eremius*), a small (∼6–8 cm), sexually-dimorphic freshwater fish endemic to the Lake Eyre Basin of Central Australia [13]. Desert gobies are found in a wide range of habitats, from permanent artesian springs to ephemeral rivers and streams. During the breeding season, males establish nests in crevices under rocks and attract passing females using colourful courtship displays that involve jerky body movements accompanied by the raising of the males' dorsal and anal fins (fin flare displays) [14]. If successful in his efforts, the female will enter the male's nest and attach her eggs in a single layer on the ceiling of the nest. Males, alone, are responsible for offspring care, with fathers tending the broods until hatching. During this time, males actively fan the brood with their pectoral fins to help ventilate the eggs and remove debris. Depending on the size of the nest, male desert gobies, like other species of egg-guarding fish (e.g. [15], [16], [17]), have the potential to receive and look after eggs from multiple females. We therefore aimed to investigate whether egg-tending males compromise care when presented with the opportunity to attract additional females. On the one hand, if parental and mating efforts are in conflict, one might predict males to reduce their level of care to pursue further mating opportunities [4]. Alternatively, if caring for offspring can also function as a signal of mate attraction, the presence of additional females should have the opposite effect, with males increasing (rather than decreasing) their parental efforts in the presence of a female [12].

## Methods

### Collecting and housing

Desert gobies were collected from the Lake Eyre Basin in Central Australia using dip and seine nets. Fish were transported back to the laboratory by 4WD in insulated 50 L plastic tubs (coolers) filled with water to a depth of 30 cm (stocking density approx. 50 fish/tub). Water in each tub was aerated using air pumps fitted to a portable electric generator. The journey back to the laboratory took two days, with partial water changes performed at the start of day 2, whereby 50% of the water in each tub was replaced with fresh, de-chlorinated tap water. The collection and transportation methods employed resulted in zero mortality.

Back in the laboratory, fish were sorted and housed in separate-sex 300 L aquaria (approx. 50 fish/aquarium) at a temperature of 24–26°C on a 12-h light∶dark cycle. Tanks were filled with water maintained at a salinity of 5‰ to mimic field conditions (using Coralife Scientific Marine Grade Salt, ESU Inc., USA). Salinity levels were monitored weekly with a Hanna H198130 conductivity meter and, if necessary, adjusted to achieve the desired concentration by adding either salt or filtered tap water to the tanks. All fish were fed daily on a diet of frozen brine shrimp (*Artemia* sp.) and commercial fish pellets.

### Experimental procedure

Experimental trials were carried out in aquaria measuring (length×width) 30×20 cm. Each aquarium was filled to a depth of 15 cm with water maintained at the same temperature and salinity as the holding tanks. A 9 cm length of PVC pipe (3 cm diameter) positioned length-wise in the middle of each aquarium served as an artificial nest. The size of the pipe was such that males could easily accommodate eggs from more than one female. Each pipe was capped at one end with the opening facing the front of the aquarium and was anchored into place by securing the pipe onto a ceramic tile buried into the substrate. The inside of each pipe was lined with a piece of transparent acetate sheet onto which a female could later attach her eggs. The use of the transparency allowed us to remove and photograph the egg mass so that we could later count the number of eggs that were laid. Removal of the acetate causes minimal disturbance to the male who quickly resumes care of his clutch once it is returned to the nest.

In order to examine whether there is a conflict between parental care and future mating opportunities, we first needed to obtain egg-tending males. To achieve this, we introduced one sexually mature male (identified by the presence of nuptial colouration) into each experimental tank. After the male had acclimated to the tank and taken up residence within his nest, a gravid female was introduced into the tank and the pair was allowed to spawn. Nests were checked twice daily (morning and afternoon) for the presence of eggs. If the original female had not spawned within a week, she was replaced with another. After spawning had taken place, we removed the female, carefully slid the acetate sheet out of the nest, and photographed the clutch. After photography, the clutch was returned to the male who was then allowed to care for the brood. Males were then randomly assigned to one of two experimental treatments, with trials commencing two days after spawning.

Depending on treatment, trials involved 20 min observations of egg-tending males in either the presence (n = 15) or absence (control; n = 15) of a new (i.e. ‘stimulus’) female. The former was achieved by placing a gravid female into a compartment created inside the front of the male's tank using a clear, Perspex divider. The female was introduced into this compartment and allowed to acclimate 10 min before the commencement of each trial. During this time, she was hidden from the male's view by covering the clear Perspex divider with a black plastic sheet. The sheet was removed at the start of the trial so that the male could see and respond to the stimulus female. To avoid potential differences in the level of disturbance between treatments, control trials were subjected to the same manipulation (i.e. use of dividers and black sheeting) except for the actual introduction of a stimulus female. Males in the two treatment groups did not differ significantly in body size (mean total length ± SE of males in ‘stimulus female’ treatment = 69.65±1.04 mm, control = 69.78±0.84; t-test: t_28_ = 0.095 p = 0.93) nor in the size of the clutch that they were tending (mean ± SE number of eggs tended by males in ‘stimulus female’ treatment = 217.93±27.97, control = 213.80±22.46; t-test: t_28_ = 0.12 p = 0.91).

For both treatments, we recorded male behaviours during the 20 min observation period using a Sony HC 96 camcorder positioned in front of the tank. These digital recordings were subsequently analysed using the computer software program Etholog. Desert gobies fan their eggs by alternatively beating their right and left pectoral fins in ‘bouts’ that last between 5 s and 1 min (personal observation). We recorded the number of fanning bouts, as well as the duration (s) of each bout. We also quantified the fanning intensity (defined as the number of fin beats per s of fanning), the total number of pectoral fin beats and the total time (s) spent inside and outside the nest. In addition to behaviours captured by the digital recordings, we conducted 10 s spot samples of courtship during the actual trials, directly observing male position and courtship behaviour (number of fin flare displays) within the aquarium (sensu [14], [18]). After completion of the experiment, adults (and any resulting offspring) were retained as stock for future unrelated research.

Statistical analyses were carried out using the statistical program R 2.10.1 [19]. We checked all data for normality and heterogeneity of error variances and applied the necessary transformations, where appropriate, prior to analyses.

This study complies with all the relevant Federal and State laws of Australia and was carried out under ethics permit no. BSCI/2007/12 from the Biological Sciences Animal Ethics Committee of Monash University.

## Results

Male desert gobies performed less fanning in the presence of a female (Table 1; t-test: t_28_ = 2.066; p = 0.048) and engaged in shorter fanning bouts (Table 1; t-test: t_28_ = 2.08; p = 0.047). The actual number of fanning bouts, however, did not differ significantly between treatments (Table 1; t-test: t_28_ = 1.78; p = 0.08) nor did we find any difference in male fanning intensity (Table 1; t-test: t_28_ = 1.1; p = 0.28).

**Table 1:** Behaviour of males in the presence (N = 15) and absence (N = 15) of a stimulus female.

	Female	
	Present	Absent
Number of fin beats	161.80±38.27	320.93±68.03
Fanning bout length (s)	13.37±3.02	20.70±3.18
Fanning bout number	10.07±2.39	14.20±1.95
Fanning intensity (beats/s)	1.484±0.200	1.719±0.074
Time outside nest (s)	380.40±117.01	30.20±28.87

Data are presented as mean ± 1 SE.

**Figure 1:**
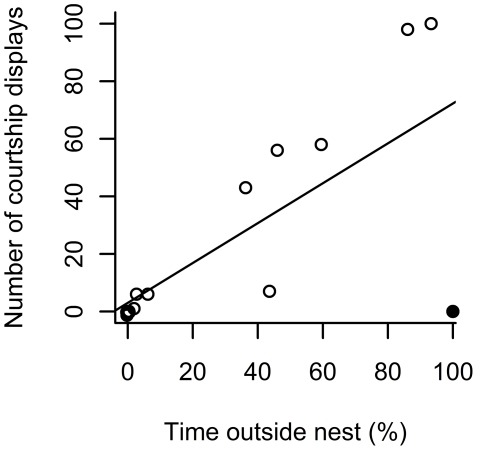
Relationship between time spent outside the nest and courtship in male desert gobies in the female stimulus trials (n = 15). The closed circle indicates an influential outlier.

Males that were exposed to a female spent significantly more time outside the nest (Table 1; Welch's t-test: t_21.0_ = 3.54, p = 0.002). Within the ‘stimulus female’ treatment there was a significant positive relationship between the time males spent outside the nest and their courtship effort (Fig. 1; linear regression R^2^ = 0.47, F_1,13_ = 13.64, p = 0.002). In this data, there was a highly influential outlier (Cook's D = 0.62, Fig. 1). Performing the analysis with this outlier removed strengthened the relationship (R^2^ = 0.90, F_1,12_ = 118.9, p<0.001).

## Discussion

Male desert gobies reduced their parental effort when presented with additional mating opportunities. More precisely, we found that males spent more time outside their nest, engaged in shorter bouts of fanning and fanned less overall in the presence of females. There was also a non-significant tendency for males to engage in fewer numbers of fanning bouts. However, the intensity of fanning (fan beats per second) was unaffected by the presence of females. Thus, males did not appear to compensate for the reduced time in the nest by fanning more intensely. Taken together, these results suggest a temporal conflict between male parental effort and mating effort in the desert goby, with males having to choose between leaving the nest to court additional females and remaining inside the nest to provide care for their clutch.

Similar trade-offs between signalling and parental effort have been observed in other species, most notably in systems with biparental offspring care [4]. For example, male fairy martins reduced their parental effort and spent more time away from the nest when the availability of fertile females were high [6], while in rainbow cichlids (*Herotilapia multispinosa*) the opportunity for extra pair copulations often led males to desert their mates who, in turn, were left to provide sole care for the brood [20]. Here, it is worth bearing in mind that in species with biparental care, investigations of male care can be confounded by the behaviour of the social mate as a result of differential allocation [21], [22], [23]. This, however, is not an issue in the current study since male desert gobies are the sole carers of their young.

Recently, it has been suggested that male parental and mating effort are less likely to come into conflict in polygynous species with male-only care, because such males are often capable of continuous breeding at the same location where males are looking after their young (e.g. nesting sites) [9]. Our results, however, suggest that temporal constraints may nevertheless be important, as males are not physically able to engage in care and courtship simultaneously. Comparable results, in this regard, have also been reported in the two spotted goby (*Gobiusculus flavescens*), with males having to reduce nest care behaviours in order to leave the nest to pursue additional mating opportunities [24]. The consequences this might have on the survival of offspring are likely to depend on their vulnerability to predators and other environmental variables [25], [26].

Intriguingly, our finding of a temporal trade off between courtship and care contrasts with that of another related species, the sand goby. In that species, males have been observed to spend more (rather than less) time in the nest and actually increase the caring behaviours they perform when potential suitors are nearby [12]. Why might this be so? Sand goby females are known to prefer males that engage in higher levels of egg-fanning behaviour [10]. In this regard, female preference for male care is expected to increase female fitness because selection for males that provide more care may result in higher egg hatching success [27]. On the other hand, the capacity for males to adjust their fanning behaviours in the presence of females could potentially also undermine the reliability of egg fanning as a signal to choosy females [8].

In fish with exclusive male care, superior parental abilities appear to be especially important in guiding female mating preferences [28]. Desert gobies inhabit harsh and unpredictable environments that are often characterised by wide fluctuations in temperature, oxygen levels and salinity [29]. Under those conditions, the quality of care provided by males is likely to have a critical influence on offspring survival. Hence, female desert gobies should benefit by selecting good carers. However, if egg fanning is subject to male manipulation (sensu [12]), female desert gobies might evolve preferences for other, more honest signals when choosing their mates, instead of relying on direct observation of male care behaviours. The actual traits used by female desert gobies in selecting potential suitors are currently unknown, but in other species of fish, male courtship (e.g. [27], [30], [31]), the presence of eggs already in the nest (e.g. [11]), the quality of the nest itself (e.g. [32]), and the size of the male's pectoral fins (e.g. [33]) have all been implicated as potential signals of superior paternal care.

In summary, we found evidence of a temporal trade-off between paternal care and mate attraction in the desert goby. The results of our study are consistent with those reported in several other species [4], [24] but also provide an interesting contrast to recent work where males were found to increase their paternal effort in the presence of females [12]. Taken together, these results suggest that patterns of male reproductive investment in care and mating can be difficult to generalise, even among closely-related taxa. Future studies would do well to consider the circumstances under which conflicts and synergies can arise among different aspects of male reproductive investment and how these, in turn, can operate to influence male mating decisions.
